# Negative electroretinograms: genetic and acquired causes, diagnostic approaches and physiological insights

**DOI:** 10.1038/s41433-021-01604-z

**Published:** 2021-06-14

**Authors:** Xiaofan Jiang, Omar A. Mahroo

**Affiliations:** 1 Institute of Ophthalmology, University College London, London, UK; 2 Retinal and Genetics Services, Moorfields Eye Hospital, London, UK; 3 Section of Ophthalmology and Department of Twin Research and Genetic Epidemiology, King’s College London, St Thomas’ Hospital Campus, London, UK; 4 Department of Physiology, Development and Neuroscience, University of Cambridge, Cambridge, UK

**Keywords:** Retinal diseases, Retina

## Abstract

The dark-adapted human electroretinogram (ERG) response to a standard bright flash includes a negative-going a-wave followed by a positive-going b-wave that crosses the baseline. An electronegative waveform (or negative ERG) results when the b-wave is selectively reduced such that the ERG fails to cross the baseline following the a-wave. In the context of a normally sized a-wave, it indicates a site of retinal dysfunction occurring after phototransduction (commonly at the photoreceptor to bipolar cell synapse). This is an important finding. In genetic disease, the pattern of ERG abnormality can point to variants in a small group of genes (frequently those associated with congenital stationary night blindness and X-linked retinoschisis, but negative ERGs can also be seen in other conditions including syndromic disease). In acquired disease, there are numerous causes, but specific features may point to melanoma-associated retinopathy (MAR). In some cases, the visual symptoms precede the diagnosis of the melanoma and so the ERG findings can initiate investigations facilitating early detection and treatment. Negative ERGs can occur in other paraneoplastic conditions, and in a range of other diseases. This review will outline the physiological basis for the negative ERG, report prevalences in the literature from different cohorts, discuss the range of causes, displaying examples of a number of ERG phenotypes, highlight features of a clinical approach to patients, and briefly discuss further insights relating to current flows shaping the a-wave trough and from single-cell transcriptome analysis.

## 摘要

负性视网膜电图: 遗传及获得性疾病的病因、诊断方法和生理解析

摘要

暗适应视网膜电图 (ERG) 对标准闪光反应包括一个负向a波和一个穿过基线的正向b波。当b波被选择性地降低, 以致ERG不能穿过a波之后的基线时, 就会产生负性波 (或负性ERG) 。在正常大小的a波背景下, 它意味着视网膜功能障碍发生在光转导之后 (通常在光感受器到双极细胞的突触部位) 。这是一个重要发现。在遗传性疾病中, ERG的负性异常改变可以指向一小群基因的变异 (通常与先天性静止性夜盲和X连锁视网膜劈裂有关, 但在其他综合征性的疾病中也可以看到负性ERG) 。在获得性疾病中, 有许多原因可以引起负性ERG, 但具体特征可能与黑色素瘤相关视网膜病变有关 (MAR) 。在一些病例中, 视觉症状的发现早于黑色素瘤的诊断, 因此ERG的发现可以尽快启动诊断和治疗。负性ERGs可在其他副肿瘤性疾病和其他一系列疾病中出现。本文将概述负性ERG的生理学基础、报告不同队列文献中的患病率、讨论病因的种类范围、展示一些典型ERG的表型病例以及突出临床治疗方法的特点, 并简要讨论了有关形成a波谷的电流和单细胞转录组分析的更多见解。

## Introduction

**Fig. 1:**
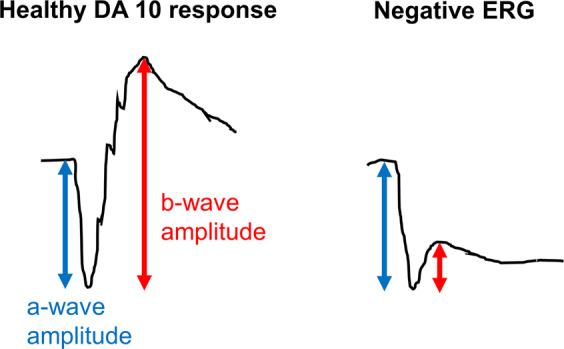
Schematic of a normal (left panel) and electronegative (right panel) DA 10 ERG response. The a-wave amplitude is measured from baseline to a-wave trough, whilst the b-wave amplitude is measured from a-wave trough to b-wave peak. A negative ERG emerges when the b-wave is smaller than the a-wave.

The dark-adapted human electroretinogram (ERG) response to full-field flashes of a range of stimulus strengths includes an initial negative-going component, the a-wave, followed by a positive-going component, the b-wave. The a-wave arises largely from hyperpolarisation of the photoreceptors in response to the flash stimulus, and the b-wave arises largely from depolarisation of ON bipolar cells (which occurs in response to the reduction in glutamate release at the photoreceptor to bipolar cell synapse). A-wave amplitudes are measured from baseline to the negative a-wave trough; b-wave amplitudes are conventionally measured from the a-wave trough to the peak of the b-wave (Fig. 1).

In response to most commonly used flash stimuli, the b-wave is larger than the a-wave in healthy individuals: this includes the DA 3 and DA 10 stimuli of the current International Society for Clinical Electrophysiology of Vision (ISCEV) standard protocol [1, 2] as well as the LA 3 stimulus; the DA 0.01 dim flash elicits a b-wave usually with a minimally detectable a-wave. If the b-wave is smaller than the a-wave, this is termed a negative ERG or an electronegative waveform (Fig. 1), and, if present in response to standard stimuli, indicates pathology [3]. This might occur in response to flashes delivered in the dark-adapted or light-adapted state, but much of this review will focus on conditions in which negative ERGs are recorded in the dark-adapted state to the standard 3 and 10 cd m^−2^ s white flash stimuli (DA 3 and DA 10).

The finding of a negative ERG is significant and guides the differential diagnosis considerably [3]. In genetic conditions, negative ERGs can narrow the list of genes likely to be associated with the disease. In acquired conditions, a negative ERG can result from an inflammatory, or autoimmune (including paraneoplastic), aetiology. Melanoma-associated retinopathy (MAR) exhibits a negative ERG phenotype often indistinguishable from that seen in complete congenital stationary night blindness (CSNB) [4], and the retinopathy can, in some cases, precede the detection of the melanoma. Tables 1 and 2 summarise genetic and acquired causes of negative ERGs.

**Table 1:** Some genetic causes of negative electroretinograms.

Ocular or systemic	A-wave normal?	Disease	Inheritance and genes	Additional clinical features
Ocular disease	Normal	Complete CSNB	X-linked: *NYX* Autosomal recessive: *TRPM1*, *GRM6*, *GPR179*, *LRIT3*	ERG features: DA 0.01 response undetectable (“completely” abolished). LA 3 has broadened a-wave trough with sharply rising b-wave. These are features of selective ON bipolar cell dysfunction. Clinical features: Patients have myopia and life-long night blindness, sometimes with reduced acuity and nystagmus
		Incomplete CSNB	X-linked: *CACNA1F* Autosomal recessive: *CABP4*	ERG features: DA 0.01 response reduced but detectable (“incompletely” abolished). LA responses severely affected, double peak in 30 Hz flicker ERG. There is both ON and OFF bipolar cell dysfunction (as site of impairment is presynaptic at the photoreceptor to bipolar cell synapse) Clinical features: Patients have myopia, variably reduced acuity and might have nystagmus. Variable history of night blindness or sometimes photobphobia
		X-linked retinoschisis	X-linked: *RS1*	ERG features: DA 0.01 response reduced but detectable. LA responses usually abnormal with 30 Hz flicker delay. Occasionally (in milder missense variants), DA 10 is not electronegative, but has reduced b:a ratio, and 30 Hz ERG might not be delayed Clinical features: variable reduction in central vision in childhood. OCT shows macular schisis. Patients may have peripheral schisis and a peripheral retinal sheen. Can be associated with vitreous haemorrhage in children. In adults, the central macula may show atrophy without schisis
	Normal or subnormal	*CRX*-associated disease	Autosomal dominant: *CRX*	Features of *CRX*-associated retinopathy are variable with family members showing variable expressivity. Some patients have a bull’s eye maculopathy and others may have wider retinal involvement. Some patients may show a negative ERG. Symptoms might occur later in life
	Subnormal	Fundus albipunctatus	Autosomal recessive: *RDH5*	ERG features: reduced DA responses (undetectable DA 0.01) with preserved LA responses. The retinoid cycle is impaired, affecting delivery of 11-*cis*-retinal to photoreceptors. DA 10 response might be electronegative reflecting the dark-adapted cone system response. Responses might normalise with prolonged dark adaptation Clinical features: life-long night blindness. Fundus shows multiple white dots. Usually non-progressive. (Variants in *RDH5* can also be associated with progressive retinal degenerations.)
		Oguchi disease	Autosomal recessive: *GRK1*, *SAG*	ERG features: reduced DA responses (undetectable DA 0.01) usually with preserved LA responses. Shut-off of activated rhodopsin is impaired and so rods lose sensitivity (as the light-sensitive current is abolished). DA 10 response might be electronegative reflecting the dark-adapted cone system response. Responses might normalise with prolonged dark adaptation Clinical features: life-long night blindness. Fundus shows a widespread sheen, which disappears following prolonged dark adaptation (Mizuo phenomenon). Usually non-progressive. (Variants in *SAG* and *GRK1* can also be associated with progressive retinal degenerations.)
		Others	Negative ERGs have been reported in other inherited retinal diseases, usually with subnormal a-waves; sometimes the negative ERG reflects the dark-adapted cone system response (e.g. with stationary night blindness associated with some dominant variants in *RHO*, *GNAT1, PDE6B*). Negative ERGs have been reported in association with variants in *GUCY2D*, *ABCA4*, *PRPH2*, *CHM*. Negative ERGs are uncommon in disease associated with most of these genes, and do not usually reflect primary inner retinal dysfunction. A family with dominant retinal dystrophy and negative ERGs in association with variants in *RAX2* has been reported	
Neuro-degeneration	Subnormal	Juvenile Batten disease	Autosomal recessive: *CLN3*	A-waves are subnormal and ERGs deteriorate to become undetectable. Vision deteriorates rapidly (symptoms begin between the ages of 4 and 8). Fundus can show a bull’s eye maculopathy with progressive degeneration. Neurological dysfunction follows and death ensues by early adulthood

A number of syndromes can also be associated with a negative ERG (see text): these include Duchenne and Becker muscular dystrophy (where vision does not appear to be affected), and a number of metabolic or neurological conditions, including multiple system atrophy. Juvenile Batten disease is listed in the table as these patients may present first to the ophthalmologist. Variants in *CLN3* can also give rise to a non-syndromic retinal dystrophy (not characterised by a negative ERG).

*CSNB* congenital stationary night blindness.

**Table 2:** Non-genetic causes of negative ERGs.

Aetiology	Disease	Unilateral or bilateral
Vascular	CRAO: negative DA ERG and reduction in LA b-wave	Usually unilateral
	Ischaemic CRVO: negative DA ERG or reduced b:a ratio	Usually unilateral
Systemic or drug toxicity	Ingestion/overdose of agents including quinine, vigabatrin, methanol	Bilateral
Direct ocular toxicity	Siderosis from an intraocular iron foreign body causes progressive ERG decline, affecting b-wave earlier than a-wave.	Usually unilateral
Autoimmune paraneoplastic	MAR gives an ERG phenotype similar to complete CSNB (selective ON pathway impairment) due to antiTRPM1 antibodies. CAR can cause both a-wave and b-wave reduction, but can also give rise to a negative ERG	Usually bilateral, but can be asymmetric
Autoimmune non-paraneoplastic	Non-paraneoplastic autoimmune retinopathy can cause both a-wave and b-wave reduction, but can also give rise to a negative ERG	Usually bilateral, but can be asymmetric
Other inflammatory	Birdshot uveitis can cause both a-wave and b-wave reduction, as well as 30 Hz flicker amplitude reduction and peak time delay. Negative ERGs occur commonly (sometimes with increased a-wave amplitude)	Unilateral or bilateral, often asymmetric
	Inflammatory or infective occlusive vasculitis can lead to negative ERGs (due to inner retinal ischaemia)	Unilateral or bilateral, often asymmetric
Nutritional	Vitamin A deficiency selectively reduces rod responses, and a negative DA ERG (with subnormal a-wave) can result, representing the dark-adapted cone system response	Bilateral

*CRAO* central retinal artery occlusion, *CRVO* central retinal vein occlusion, *MAR* melanoma-associated retinopathy, *CAR* cancer-associated retinopathy.

This review will discuss the physiological basis for the negative ERG, and then report prevalence in various published patient cohorts [5–8]. Genetic causes will then be considered, along with the proportions of families with disease associated with each of the main genes from a large genetically characterised UK-based inherited retinal disease cohort [9], together with illustrations of some key ERG phenotypes. A range of acquired causes will subsequently be presented, followed by features of a clinical approach to patients in view of the range of causes and diagnostic features. The additional value of ON–OFF ERGs [10] will be introduced and briefly discussed. Some further insights will be considered relating to current flows shaping the a-wave trough [11], findings from single-cell transcriptome data [12] and future treatments, before some final concluding remarks.

## Physiological basis

Phototransduction occurs in the outer segments of the rod and cone photoreceptors. In darkness, photoreceptors are depolarised by an inward current of cations entering through channels in the outer segment membrane. These are cyclic nucleotide-gated channels; they remain open when bound by cyclic guanosine monophosphate (cGMP). Photons of light bring about isomerisation of the chromophore (11-*cis*-retinal is converted to all-*trans*-retinal), and a cascade of molecular reactions culminate in the depletion of cGMP, which leads to closure of the cation channels and consequent hyperpolarisation of the photoreceptor, that is the cell membrane potential becomes more negative. (The reader is referred to the review of Arshavsky et al. for a detailed description of phototransduction) [13]. This hyperpolarisation contributes to the ERG a-wave [14].

Photoreceptor hyperpolarisation leads to a reduction in release of the neurotransmitter glutamate at the photoreceptor to bipolar cell synapse. Rods (which constitute the vast majority of photoreceptors in the human eye) synapse with ON bipolar cells. These cells depolarise (their membrane potential becomes less negative, or more positive) in response to the light-induced reduction in photoreceptor glutatamate release [15]. This depolarisation generates much of the b-wave of the dark-adapted flash ERG. Cone photoreceptors synapse with both ON and OFF bipolar cells. The latter hyperpolarise in response to the light-induced reduction in photoreceptor glutamate release. The hyperpolarisation of these cells contribute to the cone system a-wave [14], and their recovery also shapes the cone-driven b-wave (together with the depolarising responses from the cone-driven ON bipolar cells).

**Fig. 2:**
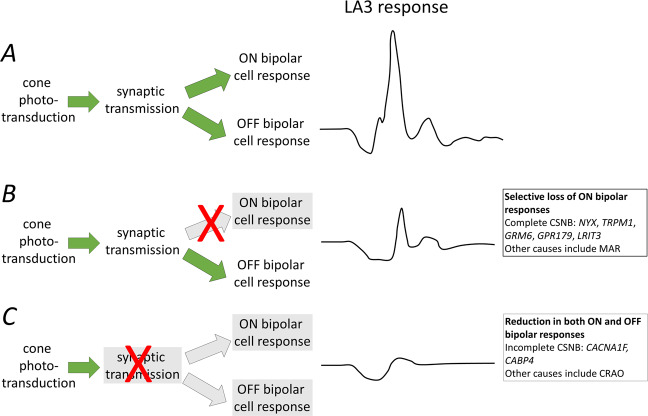
Schematic of LA 3 responses in different conditions with example causes. **A** Normal cone system signalling and normal cone system ERG. **B** In conditions with selective loss of ON bipolar cell signals, the a-wave is broadened, and the b-wave appears to be more sharply rising. **C** In conditions with loss of both ON and OFF bipolar cell signals (usually due to a presynaptic lesion), the b-wave is more attenuated. Green arrows depict normal transmission; grey arrows depict impaired transmission (grey shading indicates impairment of signal) (Color figure online).

When phototransduction occurs normally in rod photoreceptors, the dark-adapted a-wave is largely intact. Any disruption of processes after phototransduction, such as those affecting synaptic transmission or affecting the generation of the ON bipolar cell response, will selectively impair the b-wave, thus potentially resulting in a negative ERG. The genes *CACNA1F* and *CABP4* encode proteins expressed in the photoreceptor synapse involved in synaptic transmission, and so pathogenic variants result in a negative dark-adapted ERG [16]. The light-adapted (cone-driven) ERG is affected by the impairment of transmission to both ON and OFF bipolar cells. The genes *NYX*, *TRPM1*, *LRIT3*, *GRM6*, and *GPR179* encode proteins specifically involved in bringing about ON bipolar cell depolarisation; thus pathogenic variants again result in a negative dark-adapted ERG, but the light-adapted ERG exhibits a shape reflective of loss of ON bipolar cell, but intact OFF bipolar cell, contributions [16]. Figure 2 illustrates schematically changes in the light-adapted cone-driven flash ERG in these conditions. MAR gives a similar electrophysiological phenotype due to circulating autoantibodies to the TRPM1 protein expressed by ON bipolar cells [17].

Other mechanisms of disruption of post-phototransduction processes exist, including genetic, inflammatory, toxic and vascular. Due to the dual vascular supply of the retina (inner layers supplied by the central retinal artery and drained by the central retinal vein; outer layers (photoreceptors) supplied by the choroidal circulation), a central retinal artery or ischaemic CRVO will selectively affect inner retinal responses, leaving phototransduction intact, resulting in a negative waveform. Certain neurodegenerative disorders, by affecting bipolar cells earlier than photoreceptors, can also result in negative ERGs.

When the dark-adapted flash a-wave is significantly reduced, this indicates impairment of rod phototransduction. A negative ERG in this situation might in some conditions indicate additional (post-phototransduction) inner retinal disruption. In conditions where there is near abolition of rod photoreceptor light responses, the dark-adapted ERG reflects predominately the response of the dark-adapted cone system [18]. In some individuals, the dark-adapted cone system response to standard strength bright flashes is negative (i.e. the cone-driven b-wave in the dark is smaller than the cone-driven a-wave), but this is not usually observed in healthy individuals in the dark due to the simultaneously occurring, larger rod system response. It is a characteristic of cone system responses that, as flash strength increases, the a-wave amplitude increases, whilst the b-wave increases to a maximum, and then falls with further increases in flash strength (termed the “photopic hill”) [19]. The underlying mechanisms relate to changes in magnitude and kinetics of ON and OFF pathway signals [20]. With brighter flashes, the b-wave is smaller than the a-wave, yielding a negative waveform. When rod responses are highly attenuated, for example due to lack of available chromophore in Vitamin A deficiency or *RDH5*-associated retinopathy (fundus albipunctatus), or loss of rod sensitivity due to impaired shut-off of activated rhodopsin in Oguchi disease, the dark-adapted bright flash response may be negative, largely reflecting an isolated cone system response. In these cases, the a-wave is also significantly reduced [18, 21], distinguishing these conditions from those which primarily affect post-phototransduction signals.

## Prevalence of negative electroretinograms in patient cohorts

A negative dark-adapted response to standard stimuli is not usually seen in healthy individuals. In patient cohorts, negative ERGs have been reported in between 2.5% and 4.8% of those undergoing electroretinography: in studies from London, Berlin, Atlanta and Sao Paolo, figures of 4.8% [5], 2.9% [6], 4.0% [7] and 2.5% [8] have been reported, respectively. Table 3 gives the range of diagnoses found in these patients. Clearly, the overall prevalence and proportions in each category depend heavily on local clinical pathways, and the particular patients selected for electrophysiology. In a recent study from an ocular genetics service from the United Arab Emirates, 6.6% of patients had negative ERGs [22].

**Table 3:** Range of diagnoses in patients with negative electroretinograms (ERGs) from published large patient cohorts.

Diagnostic categories	Patients in each diagnosis category as a proportion (%) of all patients with negative ERGs at each centre			
	London, UK (*n* = 128) (1995–1997)	Berlin, Germany (*n* = 47) (1992–2004)	Atlanta, Georgia (*n* = 50) (1999–2008)	Sao Paolo, Brazil (*n* = 41) (2004–2013)
X-linked retinoschisis	14.8	36.2	14	7.3
CSNB	13.3	12.8	58	2.4
CRAO	10.2	0	4^a^	0
Birdshot	5.5	0	0	0
Toxic	3.9	2.1	2	0
MAR	3.1	2.1	0	0
Batten	0.8	0	0	0
Inflammatory (unspecified)	2.3	0	2	12.2
Photoreceptor dystrophy^b^	26.6	27.7	8	58.5
Multisystem atrophy	0	0	2	0
Diabetic retinopathy	0	0	0	4.9
Undiagnosed	19.5	19.1	10	14.6

Numbers of patients with negative ERGs and years reported in each study are given in parentheses.

*CSNB* congenital stationary night blindness, *CRAO* central retinal artery occlusion, *MAR* melanoma-associated retinopathy.

^a^In the row corresponding to CRAO, the patients from Atlanta included those with vasculitis as well as vascular occlusions.

^b^In patients with negative ERGs attributed to photoreceptor dystrophies, a-waves were also subnormal. This applies also to patients in some of the other diagnostic categories.

## Genetic causes

Inherited retinal diseases classically associated with negative ERG are CSNB [16, 23] and X-linked retinoschisis (XLRS) [24, 25]. CSNB has been classically divided electrophysiologically into the rarer “Riggs-type” (where there is impairment of rod phototransduction resulting in reduced a-waves) [26] and “Schubert-Bornschein CSNB” [27], where the a-wave is of normal size. This latter form will be considered in more detail here; it can further be subdivided into “complete CSNB” (associated with variants in *NYX*, *TRPM1*, *LRIT3*, *GRM6* and *GPR179*) and “incomplete CSNB” (associated with variants in *CACNA1F* and *CABP4*) [16, 23]. These conditions are associated with negative dark-adapted ERGs, with normal-sized a-waves and reduced b-waves. XLRS is associated with variants in *RS1*, and patients frequently display negative dark-adapted flash ERGs with normal-sized a-waves; in some patients the waveform may not be electronegative, but the b-wave to a-wave amplitude ratio (b:a ratio) is still subnormal [24].

**Fig. 3:**
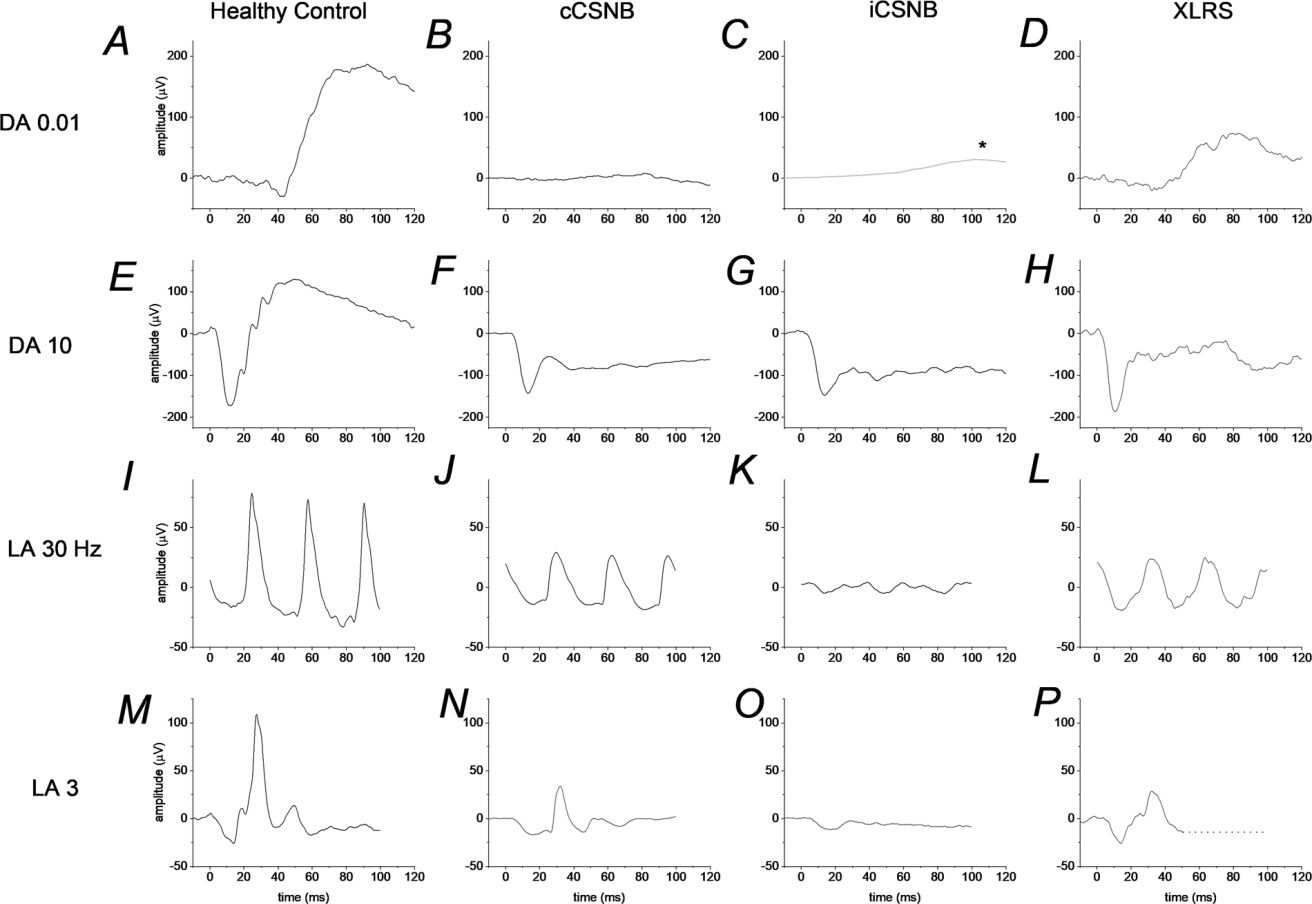
Examples of standard ERG responses in different conditions. **A**–**D** Responses to DA 0.01 stimulus (*note the response in iCSNB in **C** is schematic; the response from this patient to the dim flash was contaminated by artefact). **E**–**H** responses to DA 10 stimulus. **I**–**L** Responses to LA 30 Hz stimulus. **M**–**P** Responses to LA 3 stimulus. Responses were recorded with a conductive fibre electrode placed in the lower conjunctival fornix. The left panels show ERGs from a healthy subject. Right panels show responses from patients with complete congenital stationary night blindness (cCSNB), incomplete congenital stationary night blindness (iCSNB) and X-linked retinoschisis (XLRS) as labelled. The cCSNB patient had bi-allelic variants in *TRPM1*; the iCSNB patient had a hemizygous variant in *CACNA1F*.

**Fig. 4:**
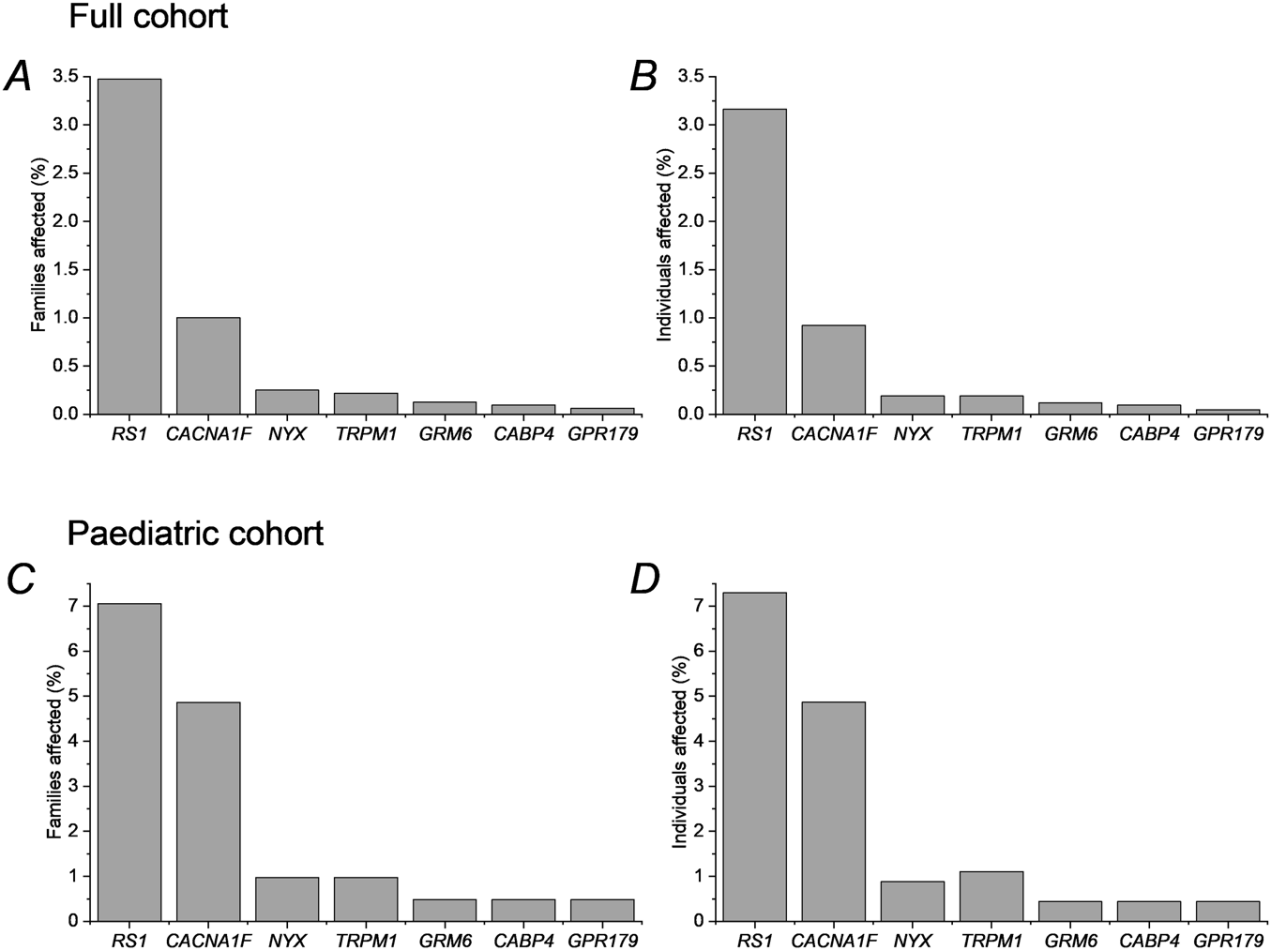
Proportions of affected families (left panels) or affected individuals (right panels) from a large genetically characterised cohort (of over 4000 inherited retinal disease patients from over 3000 families) with variants in selected genes. Upper panels  (**A**, **B**) show data for the full cohort; lower panels (**C**, **D**)  for the subset of the cohort with individuals under 18 years.

Figure 3 illustrates ERG responses to standard stimuli in XLRS and in complete and incomplete CSNB. They are described in the figure legend and when each condition is discussed in more detail below. Figure 4 gives the proportions of IRD families (and individuals) in whom disease has been associated with each of the associated genes [9]. This was from a large cohort of over 4000 IRD patients from more than 3000 families managed at a large centre in the UK (Moorfields Eye Hospital in London), in whom the genetic diagnosis was known [9]. The upper panels give proportions from the cohort as a whole and the lower panels specifically for patients under the age of 18. The proportions are higher in the lower panels, reflecting the early onset of these disorders, thus they comprise a larger fraction of the paediatric cohort. The precise proportions will be affected by current and historical testing strategies, which might generate levels of ascertainment bias; however, the figures give an approximate overview of the proportion of disease attributable to each gene.

A number of other inherited retinal diseases can be associated with negative ERGs, usually with subnormal a-wave amplitudes. In addition, some systemic conditions can be associated with negative ERGs. Table 1 summarises much of the range of genetic causes, together with associated ERG features.

**Fig. 5:**
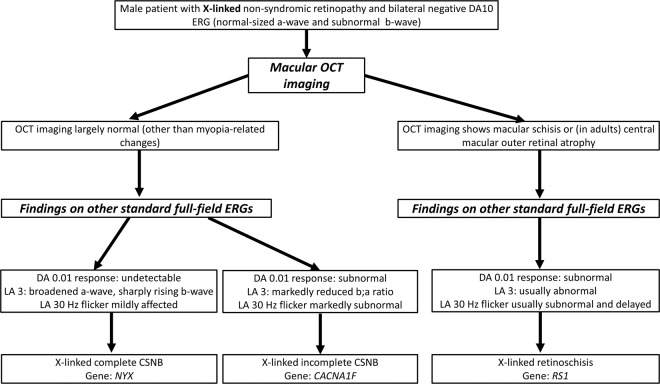
Algorithm for establishing likely associated gene in a male patient with a negative ERG and an X-linked pedigree. CSNB congenital stationary night blindness.

Three of the genes associated with negative ERGs are X-linked (*NYX*, *CACNA1F* and *RS1*); Fig. 5 presents a simple algorithm to identify the likely gene in a male presenting with a negative ERG and an X-linked family history. For a more general algorithm to identify the relevant gene in a male with non-syndromic retinopathy and an X-linked family history, the reader is referred to Figure 26 in the review by De Silva et al. [25]. In contrast to several other X-linked retinopathies, female carriers of pathogenic variants in *NYX*, *CACNA1F* and *RS1* (i.e. females with pathogenic variants on one X chromosome) do not report symptoms or display signs on fundus examination or imaging.

### Complete congenital stationary night blindness (cCSNB)

Patients with cCSNB usually have reduced visual acuity and high myopia, together with a history of night blindness. They may also have nystagmus and strabismus. Mean logMAR visual acuity has been reported as 0.3 [28] or 0.4 [29] and average refractive error has been reported as around −7 dioptres [28, 29]. Fundal examination is usually normal other than myopic changes. Standard ERG testing shows an undetectable dim flash (DA 0.01) response, and a negative ERG response to the DA 3 and DA 10 flashes, with normal-sized a-waves and subnormal b-waves. The 30 Hz flicker might be mildly subnormal and delayed, and the LA 3 response has a broadened a-wave trough with sharply rising b-wave (Fig. 3). Associated genes include *NYX* (X-linked inheritance) and *TRPM1*, *GRM6*, *GPR179* and *LRIT3* (autosomal recessive inheritance). Pathogenic variants lead to selective impairment of ON bipolar cell responses.

### Incomplete congenital stationary night blindness (iCSNB)

Patients with iCSNB also frequently display subnormal visual acuity and myopia, with nystagmus and strabismus often present. Nyctalopia, however, is not always reported, and some patients have photophobia. Mean visual acuity has been reported to be 0.4–0.5 logMAR, and average refractive has been reported as ~−8 dioptres [29] or −5 dioptres [28]. The latter study found 22% of patients to be hyperopic and also found that only 54% reported nyctalopia [28]. X-linked iCSNB is the more common form, and is associated with variants in *CACNA1F*; a rarer, autosomal recessive form of iCSNB, is associated with bi-allelic variants in *CABP4* [16], and tends to affect vision more severely. Fundal examination is largely normal other than myopic changes. However, foveal thinning has been reported in *CABP4*-associated disease [30], and inner retinal layer thinning has been reported in *CACNA1F*-associated disease [31].

ERG responses to standard stimuli show a subnormal, but not completely abolished, DA 0.01 response (helping distinguish from cCSNB). Light-adapted responses are more severely affected in iCSNB compared with cCSNB: in iCSNB, the 30 Hz flicker amplitude is severely subnormal, and may display notched, or bifid, peaks; also, the LA 3 ERG is more markedly subnormal, with a b:a ratio nearer 1, sometimes below 1.

Both genes encode proteins involved in facilitating transmission at the photoreceptor synapse. Thus, both ON and OFF bipolar cells responses are affected. As not all patients report nyctalopia, “congenital stationary night blindness” might not be appropriate. The term “cone-rod synaptic disorder”, originally proposed for disease associated with *CABP4* variants [32], might be more appropriate; this term has been subsequently applied to a range of monogenic conditions affecting presynaptic processes in signal transmission at the photoreceptor synapse, including diseases associated with *CACNA1F*, *CACNA2D4*, *CABP4* and *RIMS2* [33].

### X-linked retinoschisis (XLRS)

Patients with XLRS present with central visual impairment usually in childhood. Between 1 in 15,000 and 1 in 30,000 males are affected [34]. Visual acuity is variable, ranging in one study from 0 to 1 logMAR equivalent [35], with a more recent study reporting a range of 0.1 logMAR to no light perception, with a mean best corrected visual acuity of around 0.6 logMAR [36]. Retinal examination reveals schisis at the central macula, best seen on optical coherence tomography (OCT) imaging. The schisis in some patients might respond to carbonic anhydrase inhibitors [37, 38]. Patients may also have areas of peripheral schisis and often show a peripheral retinal sheen. Complications include retinal detachment and vitreous haemorrhage, both of which are associated with poorer visual outcomes. Older patients might show atrophic changes at the macula rather than clear schisis.

The *RS1* gene encodes retinoschisin, expressed and secreted by photoreceptors. The protein is thought to play important roles in cellular adhesion and in cell–cell interactions more generally. Roles have also been suggested in the control of ion gradients and osmolarity [39], as well as an interaction with channel proteins including *CACNA1F* [40], which could help explain some similarities in ERG phenotypes.

The DA 10 response in XLRS shows a normal-sized a-wave, with subnormal b-wave. Usually, the waveform is electronegative. Light-adapted responses are abnormal, with 30 Hz flicker delay, and often subnormal amplitudes. Patients with more severe variants (including nonsense, splice-site or frame-shifting variants) have negative DA 10 ERGs and consistently delayed 30 Hz flicker ERGs [24]. Those with milder missense variants might not show LA 30 Hz ERG delay, and the DA 10 ERG might not show a negative waveform, although the b:a amplitude ratio is usually subnormal [24]. In children with bilateral foveal schisis, the ERG is helpful in narrowing the genetic diagnosis: a negative waveform in a boy with schisis points to XLRS; a normal full-field ERG might point to other diagnoses, including *CRB1*-associated maculopathy (autosomal recessive, affecting both sexes) [41–43].

### CRX-associated disease

Pathogenic variants in the gene *CRX* (encoding a transcription factor expressed in photoreceptors) can give rise to a dominantly inherited retinal dystrophy with variable features. This can range from an isolated maculopathy, presenting later in life, to a generalised (often cone-rod) retinal dystrophy [44]. The ERG phenotype can vary, but there have been numerous reports of negative ERG waveforms in this condition [22, 45, 46].

### Other IRDs with negative ERGs

Negative ERGs have been reported in other inherited retinal diseases, but usually with subnormal a-waves. Two largely stationary conditions that involve night blindness and negative ERGs with reduced a-waves and fundus abnormalities are Oguchi disease (associated with variants in *SAG* or *GRK1*) [47–51] and fundus albipunctatus (associated with variants in *RDH5*) [18, 51]. As explained earlier, the dark-adapted responses in these conditions largely reflect the isolated cone system response; thus the mechanism underlying the negative ERG is quite different from those at play in XLRS and in complete and incomplete CSNB. Both Oguchi disease and *RDH5*-associated fundus albipunctatus are autosomal recessive and both sometimes show improvement in ERG amplitudes following prolonged (12–24 h) dark adaptation [18]. It should be noted that variants in all three genes can also be associated with non-stationary, progressive degenerations. It is possible that even typical Oguchi disease in adulthood might demonstrate slow progression [48].

**Fig. 6:**
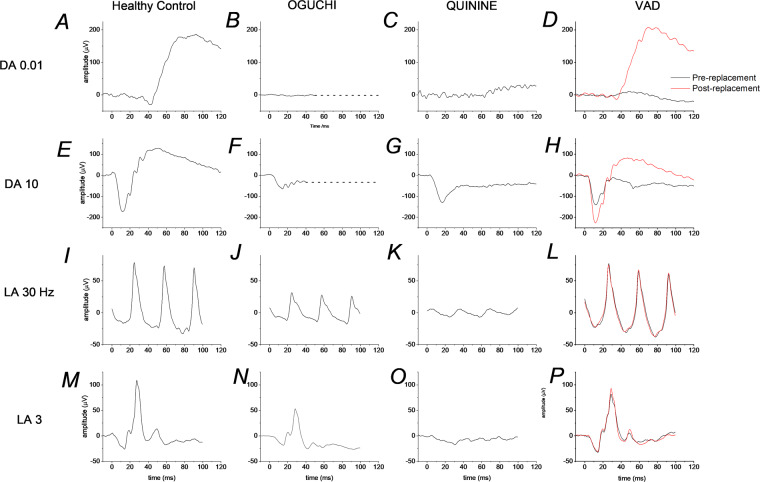
Examples of standard ERG responses in different conditions. **A**–**D** Responses to DA 0.01 stimulus. **E**–**H** Responses to DA 10 stimulus. **I**–**L** Responses to LA 30 Hz stimulus. **M**–**P** Responses to LA 3 stimulus. The left panels show ERGs from a healthy subject. Middle panels show responses from patients with Oguchi disease (associated with bi-allelic variants in *GRK1*) and prior quinine toxicity, as labelled. In the right-most panels, the black traces show responses from a patient with vitamin A deficiency (VAD); red traces show normalisation of DA responses following vitamin A replacement (by intramuscular injection) (Color figure online).

In Oguchi disease, shut-off of light-activated rhodopsin is impaired (due to defective action of rhodopsin kinase, encoded by *GRK1*, or arrestin, encoded by *SAG*). Thus, phototransduction remains active, shutting off the outer segment cGMP-gated current; the rods can no longer respond to light as the outer segment current is already abolished. Patients have a bright fundal sheen, which might disappear after prolonged dark adaptation. The sheen’s presence or absence has been associated with changes in OCT appearance of the outer retinal hyper-reflective bands [52]. After standard dark adaptation, the rods are still not electrically responsive, and so the DA 10 has a significantly reduced a-wave. The waveform is also negative as the response now reflects the dark-adapted cone system response which is often electronegative. Figure 6 shows ERGs to standard stimuli from a patient with bi-allelic variants in *GRK1*.

In fundus albipunctatus, the retina shows widespread white dots (these are subretinal deposits when examined with OCT) [18]. *RDH5* encodes a protein involved in the retinoid cycle which recycles chromophore (this cycle converting all-*trans*-retinal ultimately back to 11-*cis*-retinal via the retinal pigment epithelium (RPE)). Defects in visual cycle genes give poor night vision (as the rods are more affected by deprivation of the light-sensitive photopigment) and a low fundus autofluorescence signal, as well as subretinal white deposits [53]. In fundus albipunctatus, cone function is relatively unimpaired (cone responses are less affected by lower levels of 11-*cis*-retinal, and cones also have access to a non-RPE pathway via Muller cells) [54]. The DA ERG again may be reflective of dark-adapted cone system function (with loss of the larger rod system component), and so show subnormal a-waves sometimes with a negative waveform.

Other diseases affecting rod phototransduction (including some dominant variants in *RHO* [55, 56], *GNAT1* [57]*, PDE6B*) [58] can give negative DA ERGs due to similar mechanisms. Negative ERGs have also been reported in disease associated with variants in genes including *GUCY2D* [45], *ABCA4* [59], *PRPH2* [60] and *CHM* [61]. Negative ERGs are uncommon in disease associated with these genes, and do not usually reflect primary inner retinal dysfunction. Yang et al. also reported an autosomal dominant retinal dystrophy associated with variants in *RAX2*, in which electronegative ERGs were consistently seen [62].

### Genetic conditions with systemic involvement

Negative ERGs have been reported in a number of systemic conditions. Many of these are neurological or neurodegenerative conditions, some of which have a metabolic basis: these include disease associated with bi-allelic variants in *CLN3* (juvenile Batten disease) [63, 64], *GNB5*-associated disease [65], *WDR73* (Galloway–Mowat syndrome) [66], Spinocerebellar ataxia-1 [67] (dominantly inherited, associated with an expanded trinucleotide repeat in the gene *ATXN1*) and others. In the commonest cause of congenital disorder of glyclosylation, phosphomannomutase-2 deficiency (due to bi-allelic variants in *PMM2*), negative ERGs have been reported [68, 69]. Also, in Duchenne and Becker muscular dystrophies (associated with variants in the X-linked *DMD* gene), DA and LA flash ERGs may be negative or show a subnormal b:a ratio [70].

Of these conditions, juvenile Batten disease is perhaps the most important to highlight as the visual symptoms precede the onset of neurological dysfunction, and so these children may present to the ophthalmologist first. Vision deteriorates rapidly (symptoms begin between the ages of 4 and 8). Retinal imaging can show a bull’s eye maculopathy with progressive degeneration. Sometimes these children are erroneously diagnosed with Stargardt disease (*ABCA4*-associated retinopathy), but the visual loss is usually more profound and rapidly progressive than that seen in Stargardt disease. ERGs can be electronegative (usually with subnormal a-waves) and become undetectable [64]. Neurological dysfunction follows and death ensues by early adulthood. The diagnosis is important to make due to the important implications on patient and family counselling (including informing the parents of the risk of having further affected children). Variants in *CLN3* can also give rise to a non-syndromic retinal dystrophy (not characterised by a negative ERG) [71].

## Acquired causes

Table 2 summarises some acquired causes of a negative ERG. In a number of these conditions, the diagnosis can be made with retinal imaging or specific blood tests (or certain features in the clinical history). However, in MAR, the ERG findings are very specific [4, 17], and can lead to this important diagnosis, despite retinal imaging being near normal or showing non-specific changes. Thus, MAR will be discussed first.

### Melanoma-associated retinopathy (MAR)

This is a subset of autoimmune paraneoplastic retinopathy. In 1984, Ripps et al. [72]. reported night blindness and ERG findings resembling those seen in CSNB in a patient with a history of cutaneous melanoma. They attributed the retinopathy to the patient’s vincristine treatment. Four years later, Berson and Lessell [4] reported a patient with similar ERG findings and a history of melanoma, and concluded that the retinopathy in their patient, and in the previously reported patient of Ripps et al., represented a paraneoplastic phenomenon. Interestingly, in the same year, DuBois et al. [73] reported a negative ERG in a patient with migraine, and in a later letter, published in 1991 (in response to correspondence regarding their case) [74], they reported that the patient had informed them 2 years later that he had just been treated for an axillary cutaneous and lymph node melanoma; their earlier report was perhaps the first description of a patient in whom the visual symptoms and ERG findings preceded the diagnosis of melanoma.

The ERG phenotype in MAR is usually very similar to that seen in complete CSNB (shown in Fig. 4), reflecting selective loss of ON bipolar responses. In 2011 [17, 75], it was shown that autoantibodies to TRPM1 (expressed by ON bipolar cells, and encoded by one of the genes associated with cCSNB) [76] were present in the serum of patients with MAR, hence explaining the ERG findings. These antibodies have also been found in some patients with non-melanoma cancers, including lung [75] and ovarian [77] cancer. In patients with recent onset symptoms (which can include nyctalopia, blurring of vision, photopsia and visual field loss) and such ERG responses, investigations should be conducted for melanoma or other cancer. In patients with a known diagnosis of melanoma, onset of the retinopathy might prompt referral to oncologists to investigate for recurrence or metastasis (in the case reported by Berson and Lessell, the retinopathy preceded the diagnosis of metastasis) [4], although active retinopathy might not necessarily indicate systemic recurrence. Whilst melanoma treatment has improved dramatically in recent years, visual dysfunction due to MAR can be more challenging to treat. There have been recent reports of success of local intraocular steroid treatments [78, 79] in improving or stabilising vision.

### Cancer-associated retinopathy (CAR)

Unlike MAR, ERG findings in CAR are more variable, but can include electronegative ERGs. Such ERGs have been reported in paraneoplastic retinopathy associated with a range of cancers including lung and ovarian cancer [75, 77]. Thus in patients with such ERGs, and no obvious other cause, a systemic survey for cancer may be initiated. Autoantibodies reported in non-melanoma paraneoplastic retinopathies include antibodies to TRPM1, recoverin and others; however, several autoantibodies can also be found in non-paraneoplastic cases (see below) [80].

### Vascular causes

Retinal ischaemia or infarction secondary to retinovascular disease can lead to a negative ERG. This includes central retinal artery occlusion, ischaemic central retinal vein occlusion (CRVO) and any widespread retinal ischaemia, for example due to retinal vasculitis or extensive diabetic vascular disease. As the photoreceptors are supplied by the choroidal circulation, these are spared, and so inner retinal layer dysfunction and loss ensues. With modern retinal imaging techniques (OCT, fluorescein angiography, OCT angiography), ERGs are not usually required for the diagnosis of these conditions, although they can be helpful in showing the extent of inner retinal dysfunction. In a study of patients with diabetic vitreous haemorrhage undergoing vitrectomy, a negative pre-operative ERG was associated with poorer post-operative visual acuity, compared with those without a negative ERG [81].

### Toxicity due to systemically administered agents

Retinopathy characterised by a negative ERG waveform, can occur following administration or overdose of various agents, including quinine, vigabatrin and methanol (the latter found in antifreeze and some home-brewed alcoholic drinks); possible mechanisms have been discussed in a previous review [3]. The diagnosis should be apparent in the clinical and medication history (although direct questioning may be needed). Figure 6 shows ERG findings in a patient with longstanding quinine toxicity. Quinine has been used as an antimalarial treatment, and also for night cramps. In the past, it was used as an abortifacient. In the acute phase of quinine toxicity, patients can experience symptoms including severe visual loss, nausea, vomiting, headache and tinnitus. Fundal imaging may show retinal oedema with vascular attenuation and ERGs can show global reduction in amplitude. With time, vision may improve and a negative ERG is seen that persists [82]. OCT shows persistent inner retinal layer thinning [82].

### Direct ocular toxicity: siderosis

Intraocular iron foreign bodies can lead to retinal degeneration. They should be suspected particularly following potential exposure to high velocity penetrating injuries (when the point of entry may not be easily apparent). The inner retinal layers appear to be vulnerable to degeneration earlier than the outer retina, hence a negative ERG can result [83]. Early removal is advised to preserve or improve vision. With time, the a-wave also becomes subnormal, and the ERG can become undetectable.

### Autoimmune non-paraneoplastic retinopathies

Autoimmune retinopathies [84] are presumed to result from the action of autoantibodies to retinal antigens. They are a group of disorders with features whose variability presumably relates to numerous factors including identity of the particular autoantigens, the level (titre) of autoantibodies, and the degree to which the blood-retinal barrier is intact. Presentation is variable, usually with symptoms of photopsia or visual field defects, but can also include nyctalopia or photoaversion. Presentation can often be asymmetric or unilateral (with the second eye sometimes affected after an interval). Fundal examination may be near normal or show pigmentary abnormalities, but multimodal retinal imaging including fundus autofluorescence and OCT may reveal changes. ERGs are usually abnormal, and this can include negative ERGs. A review of cases with unilateral electronegative ERGs (that were not due to vascular occlusion or Birdshot chorioretinopathy) frequently found evidence of inflammatory changes in the affected eye, and the authors postulated an autoimmune aetiology [85]. Recently a clinical entity of acute unilateral inner retinal dysfunction has been described: the aetiology is unclear, and it is possible that inflammatory or autoimmune processes play a role [86].

Although testing is conducted for autoantibodies, their significance is not always certain, as autoantibodies to retinal antigens can be found in healthy individuals [87] and in patients with other retinal diseases, where their presence may be incidental or may be contributory to secondary destructive processes. Some patients with a diagnosis of autoimmune retinopathy can be observed initially, and treatment (which can include steroids and immunosuppressive agents) initiated in cases of definite progression; the aim of treatment is to stabilise or improve visual symptoms, or delay progression, but success is variable. An important concern is to exclude systemic malignancy as paraneoplastic disease represents a subset of autoimmune retinopathy, and presentation can be similar to non-paraneoplastic autoimmune retinopathy.

### Other inflammatory causes

Inflammatory posterior uveitic conditions can give rise to a negative ERG. A well-established association is with Birdshot chorioretinopathy (associated with HLA-A29 serotype). In these patients a variety of ERG abnormalities can be found, most consistently delay in the LA 30 Hz ERG peak time (which can be used to guide treatment) [88, 89]. Negative ERGs can be seen, and may resolve with treatment. The pathophysiology might relate to inflammatory mechanisms affecting post-phototransduction processes or the inner retinal vasculitis that some patients display. In one report, a markedly supranormal a-wave was seen, with normalisation of amplitude following treatment [88]. Other inflammatory vasculitides, particularly those leading to widespread retinovascular occlusion, can also generate negative ERGs due to inner retinal ischaemia or infarction. Infective conditions, including tuberculosis, can also give rise to occlusive vasculitis [90].

### Nutritional: Vitamin A deficiency (VAD)

Vitamin A deficiency leads to nyctalopia, but when prolonged and established, can also affect cone-mediated central and photopic vision. Other symptoms and signs may include dry skin and dry mouth, conjunctival Bitot spots (accumulations of keratin) and white dots on fundus examination (that are subretinal in location on OCT). Dietary deficiency occurs globally, but is rare in the developed world. VAD in developed countries can occur with reduced intestinal absorption, which can occur with previous small bowel disease or small bowel resection, or with liver disease (where loss of bile-production leads to reduced absorption of fat-soluble vitamins A, D, E and K). In cases of acquired nyctalopia, particularly if there is relevant medical history, VAD should be suspected and vitamin A levels checked. Vitamin A replacement in those cases where intestinal absorption is poor may need to be parenterally administered, for example by intramuscular injection.

As the light-sensitive chromophore in photoreceptors, 11-*cis*-retinal, is derived from vitamin A, VAD affects the sensitivity of photoreceptors. Rods are more numerous and more susceptible to loss of sensitivity when deprived of chromophore. Cone function is largely intact (in initial stages at least), and so night blindness is the main visual symptom. ERGs show impaired rod function, yielding subnormal DA ERG amplitudes, but relatively normal LA ERG amplitudes. The DA 3 and DA 10 ERGs will show subnormal a-waves, but may also show a negative waveform, as this may reflect the dark-adapted cone system response as discussed above [21]. Figure 6 shows ERGs in a patient with VAD. Prior to Vitamin A replacement (black traces), the DA 0.01 ERG is near undetectable, and the DA 10 ERG shows a subnormal a-wave and a negative waveform. The LA 30 Hz and LA 3 ERGs are normal. After replacement (red traces) the dark-adapted responses normalise (with increase in the DA 10 a-wave and b-wave), whilst the light-adapted responses remain unchanged.

## Clinical approach to patients

Given the range of conditions associated with negative ERGs, the clinical approach to such patients will seek to elicit key features to narrow the differential diagnosis. Electrophysiological features to consider when observing a negative ERG response to the DA 3 or DA 10 stimuli include the following: whether the abnormalities are in both eyes or just one (and if bilateral, whether they are symmetric); whether the a-wave is of normal size or subnormal; the findings in the DA 0.01 response (whether it is undetectable or subnormal, helping distinguish complete and incomplete CSNB) and the shape of the LA responses. The patient’s clinical ocular and medical history, medications, family history and results of investigations (retinal imaging and genetic investigations) are also pertinent. Table 4 summarises features of the clinical assessment. Further investigations (including systemic imaging, blood tests and gene panel testing) are guided by the findings of this assessment.

**Table 4:** Clinical approach to diagnosis of patients with negative ERGs.

Features in patient history	
Ocular history	• CSNB patients have stationary, congenital visual impairment, and are associated with myopia. XLRS usually presents in childhood. Rapidly progressive visual loss aged 4–8 can be seen in juvenile Batten disease. Acquired conditions usually have much later onset of symptoms
	• More acute/subacute onset of symptoms in adult (e.g. photopsia, night blindness) could reflect autoimmune (including paraneoplastic) disorders or inflammatory/vascular disorders. Some genetic conditions can present in adulthood
	• History of IOFB or penetrating injury may point towards siderosis
General medical history	• Specific neurological/neuromuscular/metabolic systemic diagnoses
	• Medication history to include quinine, vigabatrin (and methanol)
	• Known diagnosis or symptoms suggestive of cancer or melanoma
	• Dietary insufficiency or intestinal or liver disease that could result in VAD
Family history	• Male with X-linked pedigree suggests X-linked genes (*RS1*, *NYX*, *CACNA1F*)
	• Other generations not affected in autosomal recessive diseases (unless pseudodominance/consanguinity)
	• Dominant family history in *CRX*-related disease
Features on non-invasive retinal imaging	
Colour fundus imaging, OCT, AF	• Myopic changes in CSNB
	• Schisis in XLRS (sometimes macular outer retinal atrophy in adult)
	• Bull’s eye maculopathy and progressive degeneration in *CLN3*-associated Batten disease
	• Range of changes possible in *CRX*-related retinopathy, but symmetric
	• Sheen in Oguchi disease; small white dots in fundus albipunctatus and in VAD
	• Outer retinal abnormalities, non-specific thinning, pigmentary changes possible in autoimmune and inflammatory retinopathies, often asymmetric
	• Typical pale depigmented lesions in Birdshot chorioretinopathy. Inflammatory conditions may have cystoid macular oedema
	• Inner retinal OCT hyper-reflectivity and swelling in CRAO followed by loss of inner retinal layers over weeks months. Widespread haemorrhages in CRVO
	• Evidence of trauma or IOFB in siderosis
FFA, ICG	• FFA can delineate ischaemia/leakage in retinovascular/inflammatory disease
	• ICG can help in choroidal diseases; hypofluorescent lesions in Birdshot
Features relating to ERGs	
Are the abnormalities bilateral and symmetric?	• Genetic diseases, systemic drug toxicities, and Vitamin A deficiency should give symmetric abnormalities
	• Ocular siderosis and central retinovascular occlusions are usually unilateral
	• Paraneoplastic, inflammatory, autoimmune conditions may be unilateral or bilateral, and can be asymmetric
Is the DA 10 a-wave normal-sized or subnormal?	• Normal-sized in CSNB, XLRS
	• Normal-sized in CRAO and CRVO and certain drug toxicities
	• May be normal-sized in MAR, but can be variable in other inflammatory and autoimmune retinopathies (including CAR)
	• Can be normal or subnormal in *CRX*-related disease
	• A-wave usually subnormal in fundus albipunctatus, Oguchi disease, Batten disease and other diseases affecting photoreceptors
	• A-wave subnormal in VAD
What is the shape of the LA responses?	• Shape may reflect selective ON pathway dysfunction (cCSNB, MAR) or combined ON and OFF dysfunction (other conditions)—see Figs. 2 and 3
	• LA response may be normal when cone system function normal (and DA responses reflect intact cone function)

Features in the history and findings in non-invasive clinical investigations (including retinal imaging and ERGs) of patients that narrow the differential diagnosis. The following invasive investigations can be helpful in selected cases: fundus fluorescein angiography can delineate extent of ischaemia or leakage in retinovascular or inflammatory disease; indocyanine green angiography can further characterise choroidal disease, including demonstration of hypofluorescent lesions in Birdshot chorioretinopathy that are not evident clinically. Further investigations (including non-ocular imaging, blood tests, genetic investigations) are guided by the findings above.

*IOFB* intraocular foreign body.

## Additional ERG protocols

This review has focused on the ISCEV standard full-field ERG protocols [1, 2]. Additional non-standard protocols can be helpful in further clarifying the nature of retinal dysfunction. In particular, the photopic ON–OFF ERG can be used to help distinguish between conditions in which there is selective impairment of ON responses or combined impairment of ON and OFF responses [10]. A long flash (150–200 ms in duration) is delivered in the presence of a rod-saturating background so that responses to onset and offset of the stimulus can be separated. Conditions such as cCSNB and MAR may be expected to selectively impair the ON response. Many of the other conditions may affect both responses. In retinopathy associated with previous quinine toxicity, the ON response is markedly impaired, but there may be relative preservation of the early component of the OFF response, followed by a plateau [10]. In those conditions where cone system function is normal, these responses are also likely to be normal.

**Fig. 7:**
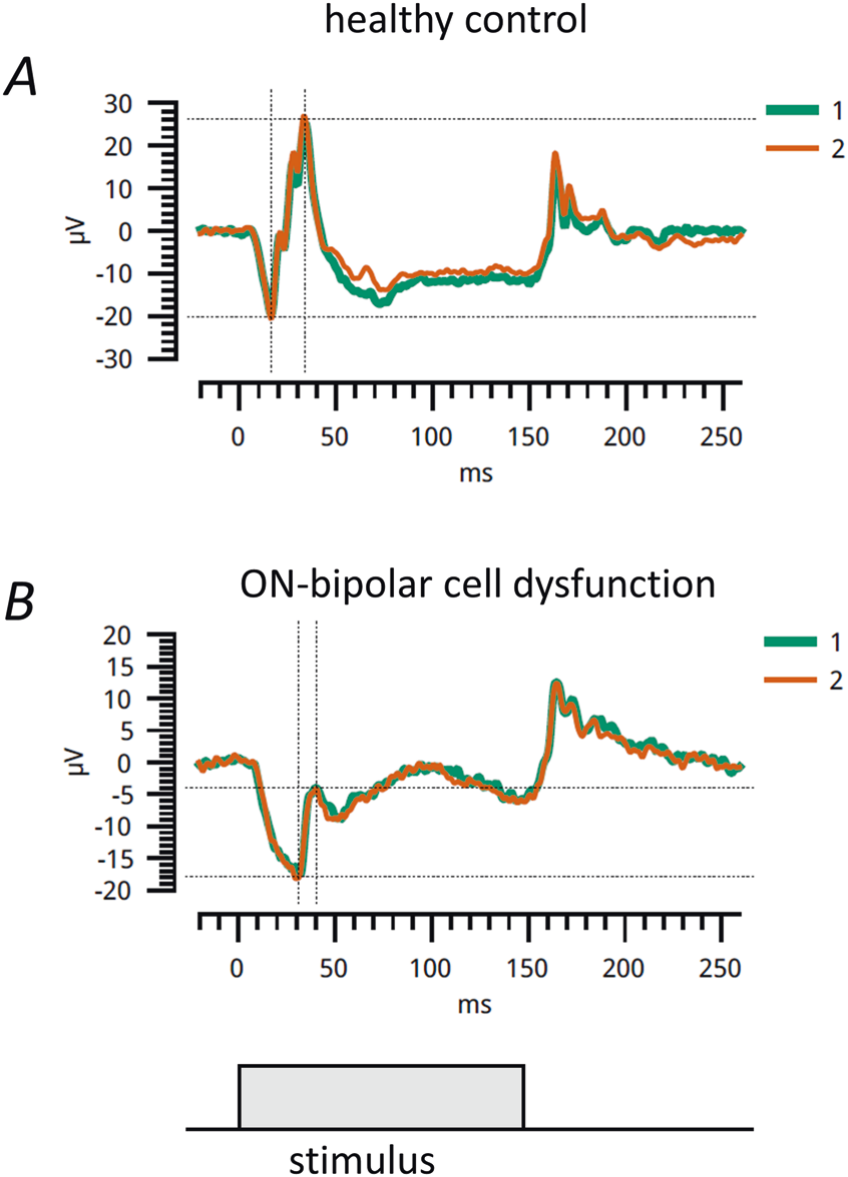
Responses to ON–OFF ERG stimuli. The stimulus is a long (150 ms) white flash delivered on a white background. The grey rectangle at the bottom shows the duration of the stimulus. **A** shows responses from a healthy individual. **B** shows responses from a patient with complete congenital stationary night blindness (bi-allelic variants in *TRPM1*). The patient’s responses show selective loss of the b-wave in response to stimulus onset. ERGs were recorded with a portable ERG device (RETeval, LKC Technologies, Gaithersburg, MD, USA).

Figure 7 shows such responses recorded with a portable device from a healthy individual: a negative a-wave followed by a positive b-wave is seen in response to stimulus onset, and a positive-going response, termed the d-wave, in response to stimulus offset. The lower panel shows the response from a patient with complete CSNB secondary to bi-allelic variants in *TRPM1*. Here, the positive b-wave response to stimulus onset is attenuated, but the response to stimulus offset appears spared.

## Further insights

### Currents shaping the a-wave trough

Several previous studies have applied mathematical models of rod photocurrent responses [91] to fit the ERG [92–94], and have assumed that the bright flash dark-adapted a-wave trough, and the immediate recovery following the trough, is due to the intrusion of depolarising currents in ON bipolar cells. Loss of ON bipolar cell depolarisation would then be expected to result in an increased a-wave amplitude. However, this appears not to be the case: patients with abolition of ON bipolar cell responses due to genetic conditions (for example complete CSNB) do not show supernormal a-waves to standard flash strengths, nor do patients with loss of inner retina following vascular occlusion. The initial recovery (repolarisation) after the a-wave trough persists (see Fig. 3). Animal models of CSNB [95], or primate recordings after pharmacological manipulation to remove post-receptoral responses [96], yield similar results. Robson and Frishman [11] have shown that the a-wave trough in response to such flash strengths (and more strong flashes) is instead likely to be shaped by current flows in parts of the photoreceptor proximal to the outer segment (in the inner segment or outer nuclear layer), and their model of these current flows appears to explain much of the available experimental and patient data. In some inflammatory conditions, a supernormal a-wave has been observed [88], and the origin of this is unclear. It is possible that the site of primary pathology is located within the photoreceptor rather than, or in addition to, the inner retina (post-receptoral layers). Modification of previous assumptions and application of newer mathematical models to patient waveforms might yield novel non-invasive quantitative assessments of retinal function in these disorders, together with newer insights into pathophysiology.

### Insights into genetic diseases from single-cell transcriptome data

**Fig. 8:**
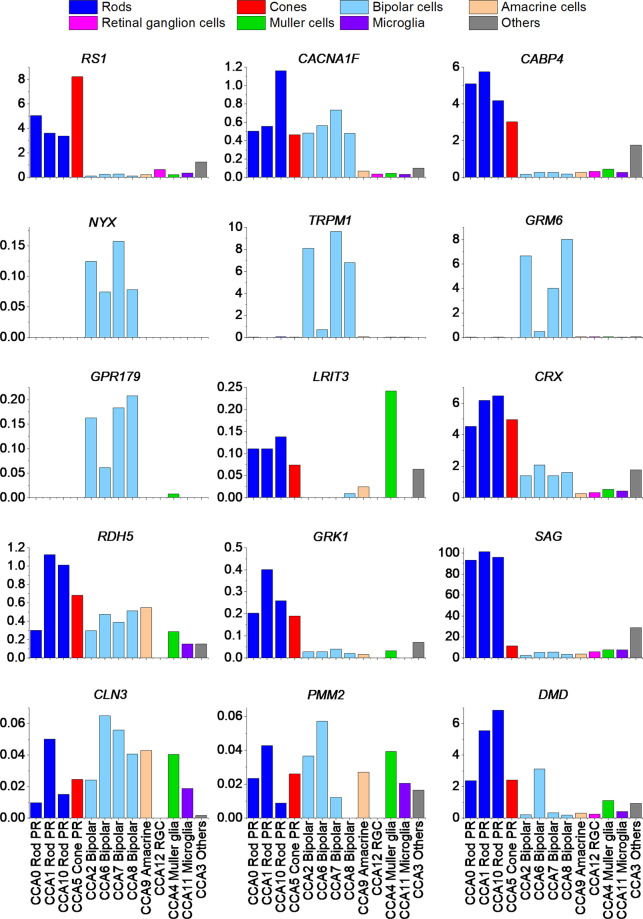
Retinal expression by cell type of genes associated with negative ERGs. Data plot expression levels found in the second supplementary table (“Dataset EV2”) from the study of Lukowski et al. (2019). This is following canonical correlation analysis to correct for batch effects. The clusters have been reordered to group cell types, and bars representing the same cell type have been given the same colour.

Although the genetic basis for several inherited causes of negative ERGs is now known, precise mechanisms of visual impairment remain to be elucidated in many of these conditions. Single-cell transcriptome data are now becoming increasingly available, and will help us understand patterns of cellular expression. A recent study reported single-cell RNA sequencing of more than 20,000 retinal cells from three human donors [12]. Several transcriptionally distinct clusters were found and the relative expression levels in different cell types are given in their supplementary material; their second supplementary dataset gives an exhaustive list of genes and expression levels following correction of batch effects by canonical correlation analysis [12]. Figure 8 plots these data for particular genes that can be associated with negative ERGs. Bars representing the same broad neuronal cell type have been given the same colour, and the clusters have been reordered so that clusters of the same broad cell type are together.

Several expected and unexpected findings emerge. The genes associated with complete CSNB are all almost exclusively expressed by bipolar cells as expected (*NYX*, *TRPM1*, *GRM6*, *GPR179*). Interestingly, *LRIT3*, although associated with a similar ERG phenotype, is expressed in photoreceptors and Muller cells with very little relative expression in bipolar cells. Thus, it might be predicted that presynaptic expression by photoreceptors is important in particular in rod to ON bipolar cell transmission. A recent murine study suggested that this is the case: presynaptic expression of *LRIT3* transsynaptically organises the postsynaptic glutamate signalling complex, which contains TRPM1 [97]. With regard to incomplete CSNB genes, although *CACNA1F* and *CABP4* both give rise to similar ERG phenotypes, Fig. 8 shows that the former is expressed in both photoreceptors and bipolar cells at comparative levels, whilst the latter is expressed very strongly in photoreceptors. It is possible that the bipolar cell expression of *CACNA1F* might mean that pathogenic variants in this gene give rise to additional impairment of transmission between bipolar cells and ganglion cells. A recent study of retinal layer thicknesses in *CACNA1F*-associated disease reported evidence of inner retinal layer thinning that appeared not explicable by the degree of myopia [31]. *CLN3* is expressed in multiple cell types, with bipolar cells showing strongest expression. This might explain the pattern of ERG degeneration with initial greater attenuation of the b-wave followed by loss of both a-wave and b-wave [64]. It should be noted that Fig. 8 only depicts expression in the neural retina; for some genes (such as *RDH5*), the RPE expression is likely to be relevant to disease.

### Future treatments

For most IRDs, there remain no medical or surgical treatments that can bring about lasting improvement of vision. A number of the non-syndromic IRDs in this review fortunately show relative stability, but some can be progressive. For *RS1*-associated disease, trials of intravitreally administered gene-replacement therapy [98] are being conducted (ClinicalTrials.gov Identifiers: NCT02317887 and NCT02416622). For *CLN3*-associated disease, a phase 1/2 open-label, single-dose, dose-escalation clinical trial of intrathecal gene therapy (ClinicalTrials.gov Identifier: NCT03770572) is listed, but has not commenced recruitment. Pharmacological treatments are also being trialled for this condition [99]. Full results of these trials will be eagerly awaited.

With respect to acquired disease, treatments for inflammatory retinopathies are improving with newer immunomodulatory agents available. In patients with melanoma, survival has improved in recent years with newer treatments, including immune checkpoint inhibitors (though these treatments have been associated with ocular side effects). Treatment of MAR, and autoimmune paraneoplastic and non-paraneoplastic retinopathy in general, remains challenging. As mentioned above, there have been anecdotal reports of success with local intraocular steroid in MAR [78, 79].

## Conclusions

Whilst progress has been made in the understanding of many of these conditions, several unanswered questions remain, both for genetic and acquired disorders (for example, the variability in phenotype in *CRX*-associated disease even within families, or why some patients develop autoimmune paraneoplastic and non-paraneoplastic retinopathies whilst others do not, even with similar cancers). The electronegative ERG is an important finding. In genetic disease, this can narrow the differential in terms of likely associated genes. In the past, genetic testing proceeded gene by gene, whereas now it is common to start with simultaneous testing of large gene panels by next generation sequencing or even to commence with whole genome sequencing. The latter strategy frequently returns numerous variants of uncertain significance. Precise evaluation or re-evaluation of the phenotype, including by electrophysiology, can help determine which variants are relevant. In the context of acquired disease, the negative ERG can point to particular diagnoses. In some cases of MAR, the visual dysfunction and electrophysiological findings might precede the melanoma diagnosis, and initiating the search for the tumour could potentially bring about early detection and treatment, with a possible influence on survival.
